# Restricting bioenergetic efficiency enhances longevity and mitochondrial redox capacity in *Drosophila melanogaster*

**DOI:** 10.1111/acel.14107

**Published:** 2024-02-11

**Authors:** Analisa L. Taylor, Olga Dubuisson, Pritika Pandey, Elizabeth R. M. Zunica, Bolormaa Vandanmagsar, Wagner S. Dantas, Alyssa Johnson, Christopher L. Axelrod, John P. Kirwan

**Affiliations:** ^1^ Integrated Physiology and Molecular Medicine Laboratory Pennington Biomedical Research Center Baton Rouge Louisiana USA; ^2^ Department of Biological Sciences Louisiana State University Baton Rouge Louisiana USA

**Keywords:** aging, BAM15, bioenergetics, mitochondrial uncoupling

## Abstract

Mitochondria are essential for survival and as such, impairments in organelle homeostasis significantly accelerate age‐related morbidity and mortality. Here, we determined the contribution of bioenergetic efficiency to life span and health span in *Drosophila melanogaster* utilizing the mitochondrial uncoupler BAM15. Life span was determined in flies fed a normal diet (ND) or high fat diet (HFD) supplemented with vehicle or BAM15. Locomotor function was determined by negative geotaxis assay in middle‐aged flies fed vehicle or BAM15 under ND or HFD conditions. Redox capacity (high‐resolution respirometry/fluorometry), citrate synthase (enzyme activity), mtDNA content (qPCR), gene expression (qPCR), and protein expression (western blot) were assessed in flight muscle homogenates of middle‐aged flies fed vehicle or BAM15 ND. The molar ratio of H_2_O_2_ and O_2_ (H_2_O_2_:O_2_) in a defined respiratory state was calculated as a measure of redox balance. BAM15 extended life span by 9% on ND and 25% on HFD and improved locomotor activity by 125% on ND and 53% on HFD. Additionally, BAM15 enhanced oxidative phosphorylation capacity supported by pyruvate + malate, proline, and glycerol 3‐phosphate. Concurrently, BAM15 enhanced the mitochondrial H_2_O_2_ production rate, reverse electron flow from mitochondrial glycerol‐3‐phosphate dehydrogenase (mGPDH) to Complex I, mGPDH, and Complex I without altering the H_2_O_2_:O_2_ ratio. BAM15 upregulated transcriptional signatures associated with mitochondrial function and fitness as well as antioxidant defense. BAM15‐mediated restriction of bioenergetic efficiency prolongs life span and health span in *Drosophila* fed a ND or HFD. Improvements in life span and health span in ND were supported by synergistic enhancement of muscular redox capacity.

## Graphical abstract

Restricting bioenergetic efficiency via BAM15‐mediated mitochondrial uncoupling improves health span and longevity in flies fed a normal or high fat diet. Enhanced health span and life span was attributed to reductions in body mass and lipid accumulation, as well as improvements in locomotor function, mitochondrial redox capacity, and the expression of genes associated with antioxidant defense and cellular fitness.

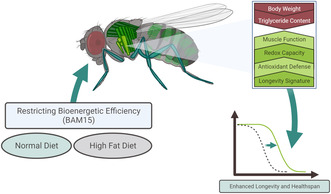

## 1 INTRODUCTION

The global population of adults over the age of 60 is expected to increase by 10% from 2015 to 2050 (Ageing and Health: World Health Organization, 2022). Aging is significantly associated with the development of major adverse conditions that diminish activities of daily living including heart disease, Alzheimer's disease, Type 2 diabetes, and cancer (Costantino et al., 2016; Hou et al., 2019). Age‐related mortality results from the exacerbation of the aforementioned diseases due to cellular and physiological decline by mechanisms that remain unclear (Kehler, 2019). Thus, there is a critical need to understand the mechanisms mediating longevity and develop therapies that promote healthy aging.

Mitochondria mediate survival through the production of adenosine triphosphate (ATP), synthesis of macromolecules such as DNA, maintenance of redox potential, and preservation of inheritance (Annesley & Fisher, 2019). Impairments to mitochondrial homeostasis significantly contribute to age‐related morbidity and mortality. Thus, mitochondria are essential organelles for maintaining cellular function (Trifunovic & Larsson, 2008). Bioenergetic efficiency, or the resulting fraction of useful energy converted to work (Liesa & Shirihai, 2013), is an important mechanism of cellular and organismal homeostasis mediated largely by mitochondria. Previously, we and others have demonstrated that restricting bioenergetic efficiency confers protection against obesity, Type 2 diabetes, cancer, and sarcopenia by improving mitochondrial fitness and cellular function in murine models (Alexopoulos et al., 2020; Axelrod et al., 2020; Dantas et al., 2022). However, the role of coupling efficiency on longevity remains largely unclear.

Here, we dissected the contribution of mitochondrial coupling control to longevity using BAM15, a mitochondria‐specific protonophore (Kenwood et al., 2014), as a model of bioenergetic inefficiency in *Drosophila melanogaster*. BAM15 treatment extended life span in flies fed a ND or HFD, which was associated with improvements in body composition and locomotor function. Mechanistically, BAM15 increased oxidative phosphorylation (OXPHOS) capacity supported by pyruvate + malate, proline, and glycerol‐3‐phosphate on ND. Concurrently, BAM15 enhanced the total mitochondrial H_2_O_2_ production rate, reverse electron transfer from mitochondrial glycerol‐3‐phosphate dehydrogenase (mGPDH) to NADH dehydrogenase (Complex I), mGPDH alone, and Complex I alone, without altering H_2_O_2_:O_2_ ratio or mitochondrial content. Taken together, these findings indicate that restricting bioenergetic efficiency contributes to maintaining physical function and maximizing longevity by maintaining mitochondrial redox capacity.

## 2 RESULTS

### 2.1 BAM15 extends life span in male flies fed a normal or high fat diet

**FIGURE 1:**
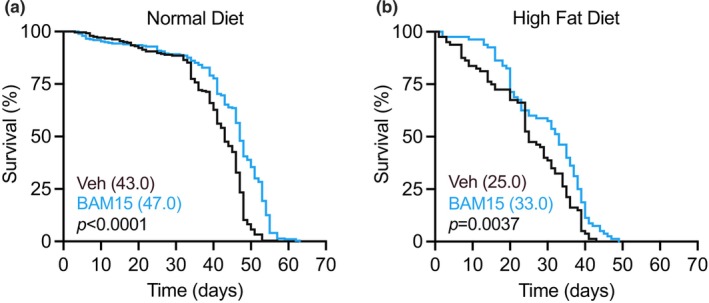
BAM15 confers life span extension in male flies under ND and HFD conditions. (a) Life span of male flies fed a normal diet (ND) or (b) a high‐fat diet (HFD) supplemented with 0.01% DMSO (Veh) or 500 μM BAM15 (BAM15). For (a), Veh *N* = 244 and BAM15 *N* = 296 flies and (b) Veh *N* = 80 and BAM15 = 80 flies included in analysis. (a) and (b) are presented as median survival and evaluated by log‐rank test.

To assess the effect of BAM15 treatment on *Drosophila* survivability, wild type Oregon R flies were fed a normal diet (ND), or high fat diet (HFD) supplemented with 0.01% DMSO (Veh) or Veh + 0.03% BAM15 (BAM15) across the life span. Under standard rearing conditions, the median survival of the wild type Oregon R strain is ~50 days (Qiu et al., 2017). The vehicle was well tolerated on ND with a median survival of 43 days (Figure 1a). HFD decreased median survival by 18 days compared to ND (Figure 1a,b). We observed that treatment with BAM15 extended life span by ~9% under ND conditions (Figure 1a), and ~24% for HFD conditions (Figure 1b). The life span‐extending effects of BAM15 was not observed in female flies (Figure S1). Taken together, BAM15 extends life span in male flies exposed to a normal diet or HFD.

### 2.2 BAM15 improves body composition and prevents the age‐related decline in climbing performance

**FIGURE 2:**
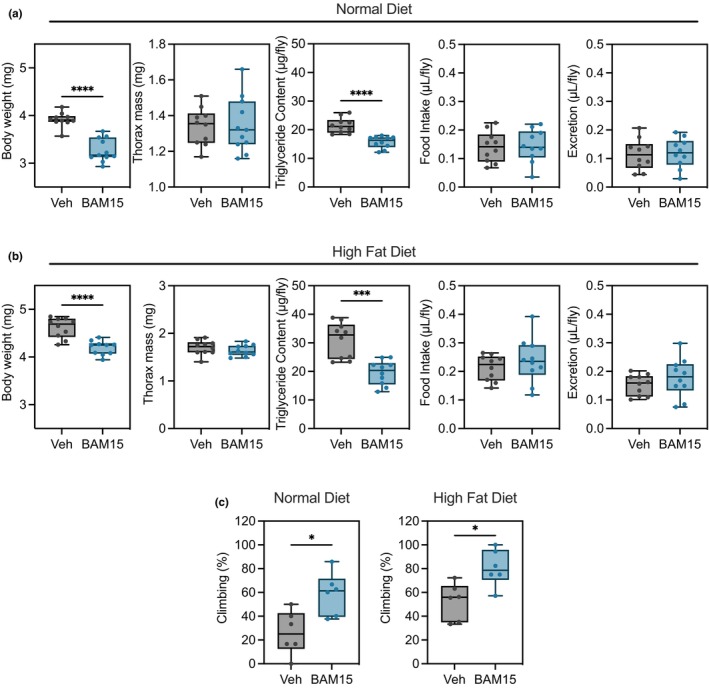
BAM15 mediates phenotypic variations in body composition and prevents age‐related decline in male fly locomotor activity in ND and HFD conditions. (a) Whole body weight (ND: Veh *N* = 10 vs. BAM15 *N* = 11; HFD: Veh *N* = 10 vs. BAM15 *N* = 10), thorax muscle mass (ND: Veh *N* = 10 vs. BAM15 *N* = 11; HFD: Veh *N* = 10 vs. BAM15 *N* = 10), triglyceride content (ND: Veh *N* = 10 vs. BAM15 *N* = 10; HFD: Veh *N* = 10 vs. BAM15 *N* = 10), and food consumption and excretion measurements (ND: Veh *N* = 10 vs. BAM15 *N* = 10; HFD: Veh *N* = 10 vs. BAM15 *N* = 10) of male flies on ND and (b) HFD. (c) Percent of flies climbing on ND at 8 weeks of age (Veh *N* = 6 vs. BAM15 *N* = 6) and HFD at 2 weeks of age (Veh *N* = 6 vs. BAM15 *N* = 6). (a)–(c) are presented as a box (mean ± 5–95% confidence interval) and whiskers (minimum to maximum) plot with all individual points and evaluated by unpaired Student's *t* test. *p < 0.05, **p < 0.01, ***p < 0.001.

We next assessed phenotypic differences by measuring whole body weight and thorax muscle mass at median life span for ND and HFD groups, respectively. BAM15 reduced body weight compared to Veh in ND and HFD fed flies (Figure 2a,b). Reductions in whole body weight were similar in ND and HFD, which was driven by decreased triglyceride accumulation without altering thorax mass (Figure 2a,b). The reduction in body fat was not explained by eating behavior or digestion, as both food consumption and excretion were similar between Veh and BAM15 (Figure 2a,b). As aging is associated with a decline in muscle mass and function (Larsson et al., 2019), we sought to determine the effect of BAM15 on locomotor activity. We hypothesized that BAM15 would rescue age‐related decline in locomotor ability in aged flies. We additionally expected HFD to exacerbate the decline in locomotor function, as chronic nutritional imbalance shortens life span and negatively impacts metabolic processes (Liao et al., 2021). Under ND, climbing performance was similar in early and midlife between groups (Figure S2). By 8 weeks of age, we observed a 125% increase in locomotor function with BAM15 relative to Veh (Figure 2c). Under HFD, a significant decline in climbing performance was observed by 2 weeks of age compared to ND‐treated flies. However, BAM15 treatment increased climbing performance by 53% in 2‐week‐old flies (Figure 2c). Taken together, these data suggest that BAM15 decreases body weight in aged flies by lowering whole‐body fat content and improved locomotor function.

### 2.3 BAM15 enhances oxidative phosphorylation capacity and synergistic H_2_O_2_ production without altering H_2_O_2_ :O_2_ ratio

**FIGURE 3:**
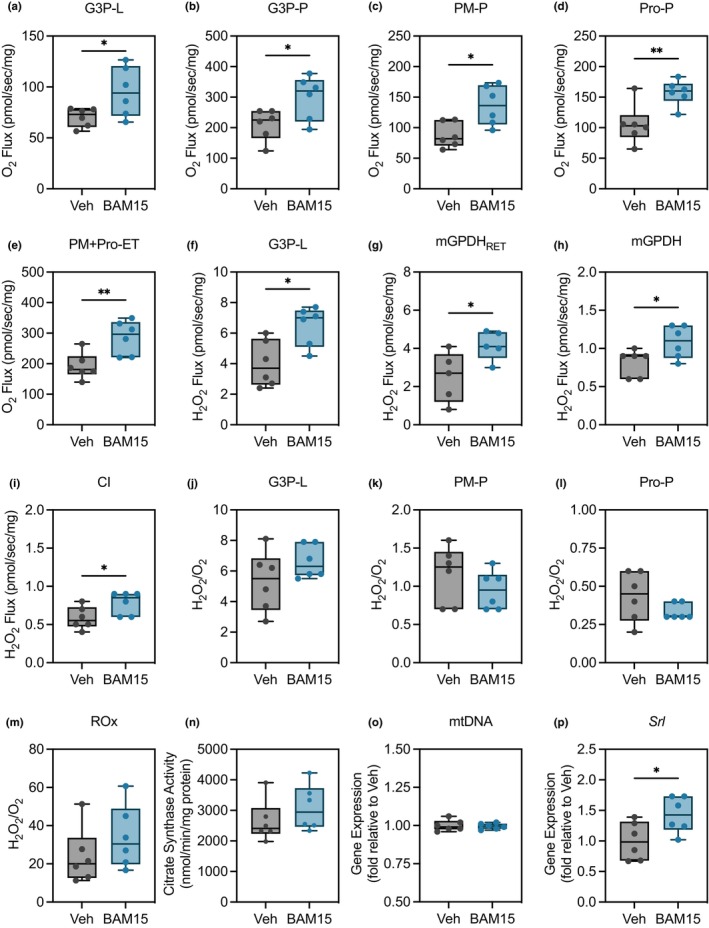
BAM15 enhances oxidative phosphorylation capacity and synergistic H_2_O_2_ production without altering redox balance or mitochondrial biogenesis. (a) O_2_ flux in the presence of G3P LEAK (G3P‐L), (b) G3P OXPHOS (G3P‐P), (c) pyruvate+malate OXPHOS (PM‐P), (d) proline OXPHOS (Pro‐P), and (e) maximal electron transfer in the presence of pyruvate+malate and proline (PM + Pro‐ET) (Veh *N* = 6 vs. BAM15 *N* = 6). H_2_O_2_ flux (f) in the presence of G3P‐L, (g) attributable to reverse electron transfer from mitochondrial G3P dehydrogenase (mGDPH_RET_), (h) of mGDPH, and (I) of complex I (Veh *N* = 6 vs. BAM15 *N* = 6). (j) H_2_O_2_/O_2_ redox balance in defined respiratory states G3P‐L, (k) PM‐P, (l) Pro‐P, and (m) residual oxygen consumption following inhibition with antimycin A (ROx). (n) Citrate synthase activity, (o) mtDNA content, and (p) Gene expression of *Spargel* (Srl). (a)–(p) are presented as a box (mean ± 5–95% confidence interval) and whiskers (minimum to maximum) plot with all individual points. (a)–(k) and (m)–(p) were evaluated by unpaired Student's *t* test. (l) was abnormally distributed and was evaluated by Mann–Whitney test. G3P, glycerol‐3‐phosphate; PM, pyruvate and malate; Pro, proline; mGPDH, mitochondrial glycerol‐3‐phosphate dehydrogenase; L, leak; P, OXPHOS; ET, electron transfer; RET, reverse electron transfer; Q, coenzyme Q; CI, complex I; Rox, residual oxygen consumption; Srl, Spargel. *p < 0.05, **p < 0.01.

Loss of mitochondrial respiratory capacity and redox poise are significant contributors to biological aging (Gorni & Finco, 2020). We therefore hypothesized that BAM15 may exert its life span and health span extending effects by enhancing oxidative phosphorylation capacity. To address this question, oxidative phosphorylation and H_2_O_2_ production were simultaneously determined across coupling control states in middle‐aged (~35 days old) male flies fed ND with Veh or BAM15 (Figure S3). We first evaluated leak respiration as a measure of quality control for the model given that BAM15's primary mechanism of action is mitochondrial uncoupling. BAM15 increased the leak of G3P relative to Veh (Figure 3a). BAM15 treatment increased OXPHOS supported by glycerol‐3‐phosphate alone (Figure 3b), pyruvate+malate alone (Figure 3c), and pyruvate+malate in the presence of proline (Figure 3d). BAM15 treatment also enhanced electron transfer capacity in the presence of pyruvate+malate and proline (Figure 3e). To assess the efflux of reactive oxygen species, superoxide dismutase (SOD) was added to convert superoxide radicals into hydrogen peroxide (H_2_O_2_), providing a net estimate of the total concentration of superoxide and H_2_O_2_ conversion. BAM15 increased H_2_O_2_ flux supported by glycerol‐3‐phosphate in the leak state (Figure 3f). We also observed increased reverse electron transfer from mitochondrial glycerol‐3‐phosphate dehydrogenase (mGPDH) to NADH dehydrogenase (CI), as evidenced by electron flow to coenzyme Q after selective inhibition of G3P with iGP‐1 and Complex I alone (Figure 3g–i). Since BAM15 enhanced both O_2_ and H_2_O_2_ flux, we sought to confirm if redox balance was shifted by calculating the H_2_O_2_:O_2_ ratio. Maintaining the balance of H_2_O_2_ and O_2_ is essential for mitochondrial function as increases in the steady‐state level of reactive oxygen species, including H_2_O_2_, due to insufficient decomposition and clearance can lead to dysregulation of cellular homeostasis and apoptosis (Georgieva et al., 2017). Surprisingly, the H_2_O_2_:O_2_ ratios were entirely intact in BAM15‐treated flies across all coupling states (Figure 3j–m), indicating that the amount of H_2_O_2_ generation occurred as a function of increasing O_2_ demand. To investigate if these differences were attributable to an overall change in the number of mitochondria, we measured citrate synthase activity and mtDNA content, biomarker of mitochondrial volume/mass, which was similar between groups (Figure 3n,o). Because uncoupling can induce mitochondrial biogenesis (Demine et al., 2019), we measured the transcriptional coactivator and master regulator of mitochondrial biogenesis, peroxisome proliferator‐activated receptor‐gamma coactivator 1‐alpha (PGC1‐α) homolog, Spargel, gene expression across samples. There was a significant increase in dPGC1‐α, suggesting a potential alteration in mitochondrial mass or activity (Figure 3p). Overall, these findings indicate a role of mitochondrial uncoupling to enhance longevity by restricting bioenergetic efficiency through increasing oxidative phosphorylation capacity and total mitochondrial H_2_O_2_ production.

### 2.4 BAM15 alters transcriptional signatures favoring enhancement of the assembly and function of the mitochondrial respiratory chain and antioxidant defense system

**FIGURE 4:**
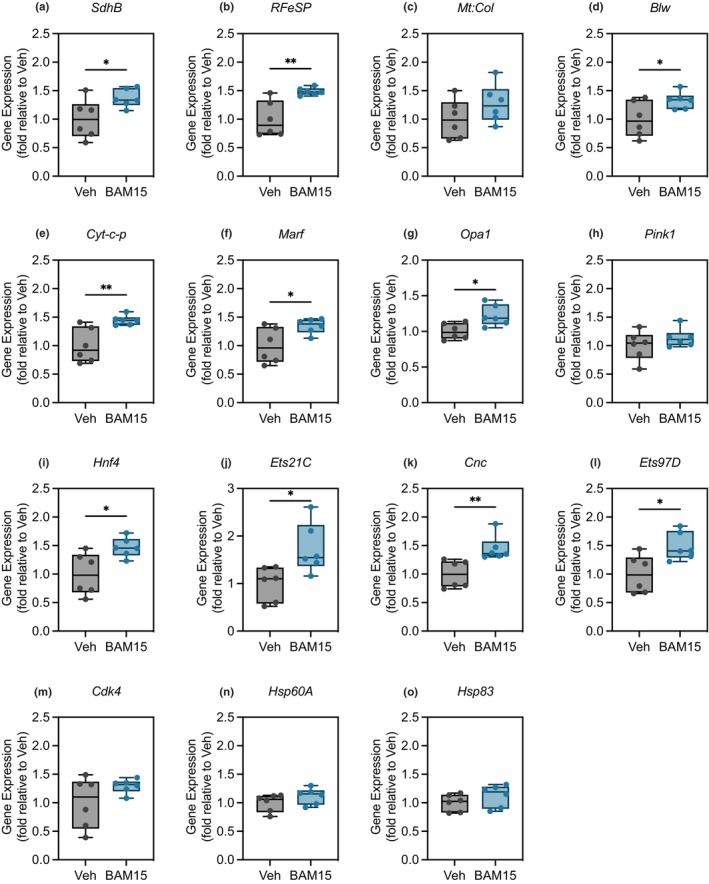
BAM15 alters transcriptional signatures favoring enhancement of the assembly and function of the mitochondrial respiratory chain and antioxidant defense system. Gene expression of (a) SdhB, (b) RFeSP, (c) Mt:Col, (d) Blw, (e) Cyt‐c‐p, (f) Marf, (g) Opa1, (h) Pink1, (i) Hnf4, (j) Ets21C, (k) Cnc, (l) Ets97D, (m) Cdk4, (n) Hsp60A, and (o) Hsp83 normalized to Gapdh1 in the flight muscle of flies treated with Veh or Bam15 on ND at 35 days of age (Veh *N* = 6 vs. BAM15 *N* = 6). (a)–(o) are presented as a box (mean ± 5–95% confidence interval) and whiskers (minimum to maximum) plot with all individual points. (a), (d), (f–l), (n), and (o) were evaluated by unpaired Student's *t* test. (b), (e), and (m) were abnormally distributed and were evaluated by Mann–Whitney test. *p < 0.05, **p < 0.01.

Given the increase in *dPGC1α*, we further assessed changes in the expression of proteins and genes related to mitochondrial function, fitness, oxidative stress, and longevity in *Drosophila* flight muscles. AMP‐activated protein kinase (AMPK), a master regulator of PGC1α activity and sensor of energetic stress, was not activated at the protein or gene level in BAM15 relative to Veh (Figure S4). *SdhB*, which encodes for the beta subunit of Complex II, was increased in BAM15 relative to Veh (Figure 4a). *RFeSP*, which encodes the rieske iron‐sulfer protein of Complex III, was increased in BAM15 relative to Veh (Figure 4b). *Mt:Col*, which encodes subunit 1 of Complex IV, did not differ between groups (Figure 4c). However, *Blw*, which encodes the alpha subunit of Complex V, was increased in BAM15 relative to Veh (Figure 4d). *Cyt‐c‐p*, which encodes the soluble cytochrome c protein requisite for OXPHOS and intrinsic apoptosis, was increased in Bam15 relative to Veh (Figure 4e). *Marf*, which encodes the *Drosophila* MFN2 ortholog required for mitochondrial fusion and assembly, was increased in BAM15 relative to Veh (Figure 4f). Similarly, *Opa1*, which mediates inner mitochondrial membrane (IMM) fusion, was increased in BAM15 relative to Veh (Figure 4g). *Pink1*, which is required for mitophagy and mitochondrial quality control, did not differ between groups (Figure 4h). *Hnf4*, which encodes a nuclear receptor protein involved in growth, reproduction, and metabolism, was increased in BAM15 relative to Veh (Figure 4i). *Ets21C*, which encodes the *Drosophila* Nrf2a transcription factor regulating antioxidant response and glutathione redox system, was increased in BAM15 relative to Veh (Figure 4j). *Cnc*, which encodes the *Drosophila* Cap n collar protein associated with Nrf2 function, was similarly increased in BAM15 relative Veh (Figure 4k). Consistently, *Ets97D*, the *Drosophila* homolog to the alpha subunit of NRF2/GABP, is increased in BAM15 relative to Veh (Figure 4l). *Cdk4*, *Hsp60A*, and *Hsp83*, which regulates cell cycle transition and heat shock response, respectively, did not differ between groups (Figure 4m–o). Taken together, restricting bioenergetic efficiency remodels the transcriptional signatures associated with mitochondrial function and fitness.

## 3 DISCUSSION

Biological aging is the result of gradual cellular maladaptation and functional decline, weakening organismal fitness, and ultimately resulting in mortality (Lopez‐Otin et al., 2013). Mitochondria contribute significantly to survival by fine‐tuning cellular energetic status, maintaining redox balance, and synthesizing macromolecules requisite for anabolic function (Vakifahmetoglu‐Norberg et al., 2017). To this end, mitochondrial dysfunction is a central tenant of biological aging (Amorim et al., 2022). Concurrently, the life span of insects and mammals can be controlled through experimental manipulation of mitochondrial function (Cho et al., 2022; Rana et al., 2017). However, the specific role of bioenergetic efficiency to health and longevity remains largely unclear. Here, we employed BAM15 as a pharmacological model of mitochondrially restricted bioenergetic inefficiency in *Drosophila melanogaster*. We found that BAM15 treatment (i) enhanced longevity; (ii) improved locomotor function; (iii) improved redox capacity relative to vehicle controls; and (iv) enhanced the expression of genes related to mitochondrial function, antioxidant defense, and longevity.

Mitochondrial uncoupling has previously been shown to prolong life span in several model organisms utilizing nonselective protonophores such as 2,4‐dinitrophenol (DNP) and FCCP (Caldeira da Silva et al., 2008; Goldgof et al., 2014; Padalko, 2005). DNP was a widely used weight loss supplement in the early 1900s but was taken off the market due to associated deaths caused by increased toxicity (Grundlingh et al., 2011). BAM15 is a recently identified small molecule protonophore exhibiting more favorable specificity to mitochondrial membranes than DNP and FCCP, which depolarize the plasma membrane (Kenwood et al., 2014). It has been previously shown that BAM15 is more potent than DNP and is orally available (Kehler, 2019), with primary distribution to liver and adipose tissue in mice (Axelrod et al., 2020; Kenwood et al., 2014). We and others have previously demonstrated that BAM15 reverses effects of diet‐induced obesity via improved metabolic conditions and increased mitochondrial respiration (Axelrod et al., 2020), reduces age‐related muscle atrophy (Dantas et al., 2022) and suppresses tumor growth and proliferation in triple negative breast cancer tumors (Zunica et al., 2021). Although these studies were previously conducted in mammalian systems, our findings support evidence for positive life and health span effects of BAM15 in a nonmammalian system and parallel the highly efficacious effects of BAM15 as observed in the previous studies. However, further studies are needed to determine if the pharmacokinetic properties of BAM15 are similar in nonmammalian organisms and ultimately whether BAM15‐mediated mitochondrial uncoupling can extend life span in mammals.

In this study, we utilized two dietary models to assess life span, including a ND that is low in fat as well as a HFD containing 15% coconut oil. Long‐term consumption of HFD increases free fatty acid concentrations above the rate of clearance (Salvestrini et al., 2019), resulting in ectopic storage of lipids and systemic adaptation of cellular processes, including alterations to genetic and epigenetic patterns, cellular exhaustion and senescence, and mitochondrial dysfunction, in a manner similar to the natural aging process (Salvestrini et al., 2019). This model of “accelerated aging” has been used widely (Galikova & Klepsatel, 2018; Liao et al., 2021) to model the effects of chronic overnutrition on life and health span. In addition to reducing life span, HFD has been shown to impair memory, behavioral responses to odor, and phototaxis memory in a manner consistent with aging (Jung et al., 2018; Rivera et al., 2019). Here, we show that mitochondrial uncoupling via BAM15 prolongs *Drosophila* life span independent of diet condition. These results build on previous findings, where BAM15 extended life span in *C. elegans* (Cho et al., 2022). We also identified phenotypic differences between BAM15 treated flies and control flies for ND and HFD conditions. Extension of life span was associated with reductions in body weight and fat mass, with no change in muscle mass. These findings are consistent with previous studies whereby BAM15 decreases plasma triglyceride and NEFA levels, and reduces fat mass in multiple mouse models (Alexopoulos et al., 2020; Axelrod et al., 2020; Dantas et al., 2022). However, in previous reports, BAM15 has been shown to increase muscle mass and cross‐sectional area, which was not observed herein (Dantas et al., 2022). This is likely attributable to model differences as *Drosophila* develop muscle weakness independent of atrophy (Piccirillo et al., 2014).

Aging confers gradual declines in physical activity due to loss of locomotor capacity and mass (Suryadinata et al., 2020). As flies age, climbing ability declines as well as other behavioral tasks that reflect age‐related locomotor impairment (ARLI) (Martinez et al., 2007). In humans, ARLI manifests as increased falling in elderly individuals, resulting in a decreased desire to be mobile (Chamberlin et al., 2005; Montero‐Odasso et al., 2005) One of the proposed mechanisms by which ARLI occurs is oxidative stress (Jones & Grotewiel, 2011), a consequence of mitochondrial dysfunction. The mitochondrial free radical theory of aging posits a role of mitochondrial oxidative stress and damage as a significant player in the development or exacerbation of biological aging and age‐related diseases (Wickens, 2001). Specifically, a build‐up of products of oxidative modification cultivates progressive mitochondrial and cellular dysfunction. Mild depolarization of the IMM has previously been shown to prevent mitochondrial ROS production, contributing to survivability (Vyssokikh et al., 2020). Here, we determined that uncoupling mediated by BAM15 synergistically enhances oxidative phosphorylation and total H_2_O_2_ production. Consistent with our findings, previous reports also show mitochondrial uncoupling mediated by BAM15 increases oxygen consumption in mice (Axelrod et al., 2020). We also show BAM15 increased H_2_O_2_ flux in various defined respiratory states. This finding was surprising, as mitochondrial uncoupling has previously been shown to attenuate oxidative stress by decreasing the amount of ROS generated by rapidly depolarizing the IMM (Caldeira da Silva et al., 2008; Vyssokikh et al., 2020). Although we found increased oxygen consumption concurrent with increased H_2_O_2_ production, the total H_2_O_2_/O_2_ redox balance was not altered between BAM15 and vehicle groups, surprisingly. We expected to see a decrease in this ratio with BAM15 treatment, consistent with the free radical theory of aging. However, this finding offers an alternative rationale, suggesting the presence of ROS could support the life span‐extending effect observed herein. In fact, previous studies show that mitochondrial uncoupling can increase ROS production with concurrent increases in antioxidant activity (Perez et al., 2009). Other evidence suggests that mitochondrial uncoupling may activate an integrated stress response (ISR) pathway to confer adaptability to stressful conditions, known as mitohormesis (Klaus & Ost, 2020). Mitohormesis relies on ROS to modulate cellular signaling and cellular health as signaling molecules (Spencer & Engelhardt, 2014). Previous studies have shown ROS to activate downstream signaling cascades to promote longevity via mitohormesis (De Haes et al., 2014). As such, BAM15 may utilize ROS as signaling molecules for cellular communication as an adaptive stress response activated either throughout life span or at a certain timepoint to prevent age‐related decline in cellular function and homeostasis. Here, we show that BAM15 treatment results in an increase in *Cnc*, encoding for the *Drosophila* Nrf2 transcription factor. Nrf2 plays a fundamental role in regulating redox homeostasis. Under oxidative stress conditions, Nrf2 is activated to regulate expression of many target genes with antioxidant activity (Kasai et al., 2020). During aging, Nrf2 expression declines concurrent to the rise in oxidative stress (Schmidlin et al., 2019). In addition to its redox homeostatic activity, Nrf2 is also responsible for regulating many mitochondrial processes, including mitochondrial biogenesis and OXPHOS (Holmstrom et al., 2016). Consistent with this, our results show an increase in *Spargel*, a *Drosophila* gene encoding for PGC1‐α. As a master regulator of mitochondrial biogenesis, PGC1‐α interacts with Nrf2 and promotes mitochondrial biogenesis and mtDNA replication via its activation (Choi et al., 2017). Additionally, H_2_O_2_ and other ROS sources drive the activation of Nrf2 via inhibition of negative regulators (Zhang et al., 2015). As such, treatment with BAM15 may be inducing a mitohormetic response necessitating the activation of Nrf2 and PGC1‐α, driving increased biogenesis and antioxidant activity. In contrast, we did not observe a change in citrate synthase activity or mtDNA content, which may indicate that *Srl* expression may not have stimulated biogenesis per se but rather increased the activation of mitochondrial gene targets (Tiefenbock et al., 2010). Maintaining optimal mitochondrial formation and maintenance is fundamental to cellular and organismal homeostasis, driven by processes such as fusion/fission. The dynamic nature of mitochondria permits interchangeable signals and communication reliant on the ability of these organelles to undergo fusion and fission. Our results demonstrate increased *Opa1* and *Marf* expression, responsible for mitochondrial fusion. This is in line with previous studies, whereby increased fusion resulted in increased longevity in *C. elegans* (Chaudhari & Kipreos, 2017). We also observed an overall increase in genes encoding ETC complexes likely contributing to the increased OXPHOS activity observed.

We also noted an increase in gene expression levels of *Hnf4*, a transcription factor responsible for regulating gene activity related to glucose homeostasis and oxidative phosphorylation (Barry & Thummel, 2016). Hnf4 is required to regulate gluconeogenesis and lipid export which becomes pronounced during periods of fasting and/or starvation. Consequentially, loss of Hnf4 increases carbohydrate dependence and impairs mitochondrial β‐oxidation, rendering fatty acid transport into the mitochondria deficient (Barry & Thummel, 2016; Palanker et al., 2009). Interestingly, it has been reported that administration of an Hnf4 agonist in conjunction with a HFD led to marked increases in mitochondrial mass, along with increased expression of mitochondrial ETC proteins in mice (Veeriah et al., 2022). Additionally, it has been reported that Hnf4 protects against ROS production (Darsigny et al., 2010). Considering the remodeling of the transcriptomic features observed in this study with BAM15, specifically targeting the regulation of mitochondrial activity and function, it is plausible that the produced H_2_O_2_ coordinates cellular communication, leading to the activation of genes such as Hnf4 and Nrf2, resulting in increased mitochondrial mass and function. Our results suggest that transcriptional remodeling induced by bioenergetic efficiency restriction favors increased lipid metabolism and longevity.

In summary, our findings indicate that mitochondrial uncoupling by BAM15 confers life span extension, improves body composition, and protects against age‐related decline in locomotor activity in *Drosophila*. Improvements in life and health span were explained, in part, by enhanced thorax redox capacity as observed by simultaneous increases in oxygen consumption and H_2_O_2_ production, as well as alteration of key transcriptional targets associated with mitochondrial activity and function following BAM15 treatment. Collectively, these data support an emerging role for restricting bioenergetic efficiency to maintain mitochondrial redox fitness across the life span.

### 3.1 Limitations

Male flies were used to exclude effects of nutrition and reproduction which potentially confound differences in life span. As such, further studies are needed to determine sex‐specific differences in response to BAM15 treatment. *Drosophila melanogaster* is a convenient model system for evaluating longevity as the median life span is 2–3 months compared to 2–3 years in mice. Future studies are required to confirm if BAM15‐mediated mitochondrial uncoupling extends life span in mammalian systems. Observation bias may have influenced the study outcomes. However, blinded independent observers were employed to minimize bias and improve reproducibility.

## 4 METHODS

### 4.1 Fly media

Male *Drosophila melanogaster* Oregon R wild type flies were housed in vials (25 × 95 mm) with a normal, macronutrient balanced diet using standard medium containing 6% cornmeal (w/v) 1.5% yeast (w/v) 1% agar (w/v), 8% molasses (v/v), 0.8% tegosept (v/v), 0.24% propionic acid (v/v), 0.02% phosphoric acid (v/v), and 0.01% dimethyl sulfoxide (DMSO) (v/v). For HFD, normal diet was supplemented with 15% coconut oil (v/v). 0.036% w/v BAM15 was added to media by dissolving in DMSO to ensure homogeneity for a final concentration of 500 μM.

### 4.2 Life span analysis

Newly enclosed flies were allowed to mate for 2–3 days on normal diet to reach sexual maturity. Male flies were then separated from female flies and dispensed into groups of 35–40 flies per vial. Flies on normal diets were maintained in polypropylene vials (VWR, cat#75813), while flies on HFDs were maintained in K‐resin vials (VWR, cat#75813), which provides a better grip surface in the oily food environment. Flies were maintained in a temperature controlled (25°C) incubator with a 12‐h light/dark cycle. Groups were fed a normal diet (ND), ND + BAM15, HFD, or HFD + BAM15 diet for the duration of the life span. Deaths was scored every 1–2 days upon transferring live flies into fresh vials. Statistical significance of the difference between survival curves was evaluated by a log‐rank test using OASIS software (Han et al., 2016). Since life span was dramatically altered by diet, middle‐age for *Drosophila* on each diet was determined as the average life span of the cohort divided by two of the vehicle‐treated group per diet. Because of the greasy nature of the HFDs, all HFD vials were maintained on their side for the duration of the life span experiments to prevent flies from getting stuck in the food.

### 4.3 Fly weights

Flies were flash‐frozen at median life span for each dietary condition for whole body weight and thorax mass measurement. Flies were thawed in PBS, botted on filter paper, and weighed in groups of five. Thorax mass was determined by removing the head, abdomen, wings, and leg segments, isolating the thorax of five flies in ice‐cold BIOPS, blotting dry on filter paper, and were weighed in groups of five.

### 4.4 Negative geotaxis

Negative geotaxis was measured to assess climbing performance as described previously (Wall et al., 2021). Briefly, 10 male flies/biological replicate were transferred to an empty, clean 100 mL glass cylinder (25 × 95 cm) and allowed to climb to the top of the cylinder after tapping to ensure all the flies started at the bottom. Percent climbing was determined as the percentage of flies that successfully traveled to the 100 mL mark of the cylinder after 1 min.

### 4.5 Triglyceride content measurements

Triglyceride content was assayed to estimate whole body fat mass by commercially available assay (Sigma MAK266) as described previously (Axelrod et al., 2023). Briefly, five flies per replicate were decapitated, weighed, homogenized in ethanolic KOH, and stored at −80°C until the time of assay. At the time of assay, samples were treated with 2 μL lipase, 25 μL of sample was added per well, and incubated for 60 min at room temperature in the dark. Absorbance was read at 570 nm (A570) and fluorescence at λ_ex_ = 535/λ_em_ = 590 nm to exclude the influence of residual eye pigment on triglyceride detection (Al‐Anzi & Zinn, 2010). Data are expressed as the triglyceride content per fly.

### 4.6 Food consumption and excretion measurements

Food consumption experiments were performed as previously described (Shell et al., 2018). Briefly, Blue #1 dye (FD and C Blue #1, Spectrum Chemical, cat# FD110‐25GM) was dissolved into either ND or HFD agar food at a concentration of 1% (w/v). Four milliliters of food was dispensed into feeder caps (MOCAP, FCS.813NA1). Three to 5‐day‐old male flies were transferred into empty K‐resin narrow fly vials (15 flies/vial) and the feeder caps were placed on top of the vials. For HFD vials, vials were placed on their side since the HFD food makes the sides of the vials greasy and prevents flies from climbing to the top of the vials. Flies were allowed to consume food for 24 h. After 24 h, flies in each vial were collected and homogenized into 1.5 mL of water, the debris was pelleted, and the supernatant was transferred to a fresh tube (internal dye, INT). The dye excreted in the vials was collected by washing the tubes with 3 mL water (excreted dye, ExVial). Note, any vials with dead flies were censored. The absorbance at 630 nm for the INT and exVial was measured for each replicate using a UV–VIS spectrophotometer. Absorbances were converted to volumes by generating a standard curve using pure dye. Total consumption was measured by combining INT + ExVial.

### 4.7 Oxidative phosphorylation and H_2_O_2_ production measurement

Oxidative phosphorylation (OXPHOS) and hydrogen peroxide (H_2_O_2_) production were simultaneously measured using high‐resolution respirometry coupled to fluorometry (Oxygraph 2 k, Oroboros) in middle aged male flies (35 days) fed ND. Middle‐aged flies were selected to avoid effects of development on redox capacity and mitochondrial function (Ferguson et al., 2005). Flies were chilled in ice‐cold BIOPS [50 mM K ± MES, 20 mM taurine, 0.5 mM dithiothreitol, 6.56 mM MgCl_2_, 5.77 mM ATP, 15 mM phosphocreatine, 20 mM imidazole (pH 7.1) adjusted with 5 N KOH at 0°C, 10 mM Ca‐(ethylene glycol‐bis(β‐aminoethyl ether)‐N,N,N′,N′‐tetraacetic acid) (EGTA) buffer, 2.77 mM CaK_2_EGTA plus 7.23 mM K_2_EGTA, and 0.1 mM free calcium] prior to isolating thorax section (Doerrier et al., 2018). The mass of isolated thorax was measured in groups of 3 prior to being transferred to a chilled dounce homogenizer containing 1 mL mitochondrial respiration medium (MiR05), [110 mM sucrose, 60 mM potassium lactobionate, 0.5 mM EGTA, 3 mM MgCl_2_‐6H_2_O, 20 mM taurine, 10 mM KH_2_PO_4_, 20 mM (4‐(2‐hydroxyethyl)‐1‐piperazineethanesulfonic acid; HEPES), and 2 mg/mL Bovine Serum Albumin (BSA), pH = 7.1], and homogenized on ice. A final concentration of 1.4 mg/mL thorax homogenates per 1 mL respiration buffer was added to the 2‐mL chambers. To determine ADP stimulated respiration initiated by complex I, glycerol‐3‐phosphate dehydrogenase (mGDPH), and proline dehydrogenase, the following substrates were injected sequentially: 15 mM glycerol‐3‐phosphate (G3P), 2.5 mM ADP, 20 μM iGP‐1 (mGPDH inhibitor), 2.5 mM pyruvate and 2 mM malate, and 5 mM proline. To determine maximal electron transfer, ~5–20 μM FCCP was injected. To determine the non‐mitochondrial residual oxygen consumption, 0.375 μM rotenone and 2.5 μM antimycin A were added to the chambers. To determine H_2_O_2_ production, Amplex UltraRed was used as an extrinsic fluorophore accompanied by the Fluorescence‐Sensor Green (Oxygraph 2k, Oroboros) external sensor to directly measure the change in fluorescence intensity indicated by the emitted wavelength. To determine H_2_O_2_ production attributed to complex I, mGPDH, and proline dehydrogenase, the following were added prior to adding G3P: 15 μM diethylenetriaminepentaacetic acid (DTPA), 10 μM Amplex Red, 1 U/mL horseradish peroxidase (HRP), 5 U/mL superoxide dismutase (SOD) and 0.1 μM H_2_O_2_. 0.1 μM H_2_O_2_ was also added in between each substrate, uncoupler, and inhibitor to calibrate the system to account and correct for decreased sensitivity of H_2_O_2_ over time. H_2_O_2_ flux attributable to G3P‐L was calculated as the H_2_O_2_ rate in the presence of G3P corrected by the background H_2_O_2_ rate. H_2_O_2_ flux attributable to mGPDH_RET_ was calculated as the ADP‐sensitive G3P‐L rate. H_2_O_2_ flux attributable to mGPDH alone was calculated as the iGP1‐sensitive G3P‐P rate.

### 4.8 Citrate synthase measurement

Citrate synthase activity was determined in flight muscle tissue homogenates using a commercially available colorimetric assay (Sigma‐Aldrich, St. Louis, MO, USA) according to the manufacturer's instructions. Briefly, 1 mL of excess tissue homogenate from the oxidative phosphorylation studies was aliquoted and samples were spun down at 5000 × ***g*** for 10 min at 4°C. The supernatant was removed, and the remaining pellet was stored at −80°C until time of assay. Frozen flight muscle pellets were homogenized in 50 μL of ice‐cold CelLytic MT Cell Lysis Reagent (Sigma‐Aldrich) using 10 strokes of a handheld homogenizer and subsequently incubated on ice for 15 min for complete tissue lysis. The lysates were centrifuged at 20,000 × ***g*** for 10 min at 4°C. The supernatant was then transferred to a chilled fresh tube and protein content was assessed by BCA assay (Thermo Scientific). Eight microliters of lysate was added to 1x assay buffer containing 30 mM acetyl CoA and 10 mM DTNB for a total volume of 190 μL and plated in duplicate on a 96‐well plate. Absorbance was measured on a plate reader set to kinetic mode (412 nm, 1.5 min, 10‐s intervals) before and after the addition of 10 mM oxaloacetate. Data were normalized to protein content.

### 4.9 RNA isolation, cDNA synthesis, and quantitative reverse transcription PCR (qRT‐PCR)

Male flies were collected at 35 days of age in groups of 25, snap‐frozen in liquid nitrogen (LN2), and placed in ice‐cold RNAlater solution on a petri dish (Thermo Fisher Scientific) to mechanically isolate thorax muscle under a dissection microscope. Isolated thorax muscles were subsequently homogenized in TRIzol™ and total RNA was extracted according to the manufacturer's instructions. cDNA was generated by using High‐Capacity cDNA Reverse Transcription Kit (Thermo Fisher Scientific) and normalized to 25 ng for quantitative real‐time PCR (qRT‐PCR) using Power SYBR Green PCR Master Mix on QuantStudio 5 real‐time PCR system. Target gene expression was normalized to *Gapdh1*. Primer sequences are detailed in Table S1.

### 4.10 Mitochondrial DNA content

Mitochondrial DNA content was determined as described previously (Zunica et al., 2021). Briefly, 15 thoraces per biological replicate were isolated, suspended in PBS (pH 7.2; 50 mM potassium phosphate, 150 mM NaCl), and homogenized. Endogenous nucleases were removed via incubation in proteinase K, followed by ethanolic extraction and elution in a mini spin column (Qiagen, Cat # 69504). Total DNA content was quantified (NanoDrop), normalized to 10 ng of DNA, and assayed by qPCR for the mitochondrial DNA to nuclear DNA ratio with primers targeting *Mt:Col* and *Cdk4* (Table S1).

### 4.11 Statistical analysis

GraphPad Prism 10 (GraphPad Software, Inc.) was used to perform statistical analyses with exception to survival which was evaluated using OASIS 2. Two group comparisons were made using Student's unpaired *t* test. Distributional assumptions were evaluated by the *F* test. If the variances were unequal, comparisons were made using the Mann–Whitney test. A log‐rank (Mantel–Cox) test was used to compare survival curves. Null hypotheses were rejected at α < 0.05.

## AUTHOR CONTRIBUTIONS

*Conceptualization*: ALT, CLA, and JPK. *Formal analysis*: ALT, OD, PP, ERMZ, AEJ, and CLA. *Investigation*: ALT, OD, PP, ERMZ, BV, WSD, and AEJ. *Resources*: AEJ and JPK. *Writing—original draft*: ALT, ERMZ, and CLA. *Writing—reviewing & editing*: All authors. *Funding acquisition*: JPK, AEJ, WSD, and ERMZ. *Supervision*: CLA and JPK.

## FUNDING INFORMATION

This research was supported in part by the National Institutes of Health grant U54GM104940 (JPK), R35GM138116 (AEJ), T32AT004094 (ERMZ), and K99AG083239 (WSD).

## CONFLICT OF INTEREST STATEMENT

The authors report no conflicts of interest related to this work.

## Supporting information


Appendix S1:

## Data Availability

Primary data will be made available upon reasonable request to the corresponding authors.
