# Supplementary material for: Restricting bioenergetic efficiency enhances longevity and mitochondrial redox capacity in Drosophila melanogaster
Source: Aging Cell. 2024 Feb 11;23(5):e14107. doi: 10.1111/acel.14107 (PMC11113268; doi:10.1111/acel.14107)
Supplement: Supplementary file 1 — Appendix S1: [file ACEL-23-e14107-s001.docx]

**Supplementary Information**

**Restricting Bioenergetic Efficiency Enhances Longevity and Mitochondrial Redox Capacity in Drosophila Melanogaster**

Analisa L. Taylor^1^, Olga Dubuisson^2^, Pritika Pandey^2^, Elizabeth R.M. Zunica^1^, Bolormaa Vandanmagsar^1^, Wagner S. Dantas^1^, Alyssa Johnson^2^, Christopher L. Axelrod^1^*, and John P. Kirwan^1^*

^1^Integrated Physiology and Molecular Medicine Laboratory, Pennington Biomedical Research Center, Baton Rouge, LA, USA

^2^Department of Biological Sciences, Louisiana State University, Baton Rouge, LA, USA

Appendix

Supplementary Table 1 – List of primers used for qPCR

Supplementary Figure 1 – Effects of Veh vs. BAM15 treatment in female flies

Supplementary Figure 2 – Climbing performance in 4- and 6-week-old flies.

Supplementary Figure 3 – Schematic illustration of substrate, uncoupler, inhibitor, titration protocol and representative high-resolution respirometric and fluorometric tracings

Supplementary Figure 4 – AMPK protein and gene expression

**Supplementary Table 1**

| **Gene symbol** | **Gene name** | **FlyBase ID** | **Forward Primer Sequence** | **Reverse Primer Sequence** |
| --- | --- | --- | --- | --- |
| dm Gapdh1 | Glyceraldehyde 3 phosphate dehydrogenase 1 | FBgn0001091 | TGAACGGCCAGAAGATCAC | ACATACTCGGCTCCAGCACT |
| dm Ets97D | Human ortholog GABPA_GA binding protein transcription factor subunit alpha | FBgn0004510 | AGCCGGACGACATCATCATC | GGCACACTCCTATCTTTTGCTC |
| dm Hnf4 | Hepatocyte nuclear factor 4 | FBgn0004914 | GCTGCAAAGGATTCTTCAGG | CTTGTCCACAACGCAGTTTC |
| dm RFeSP | Rieske iron-sulfur protein (Complex III protein) | FBgn0021906 | ATCGAGACCGAGCGAAATGT | ACACTCCGATGACCACCAG |
| dm Blw | Bellwether, (ATP5a), a protein of Complex V | FBgn0011211 | GCTCAAGCAGGGTCAGTACG | CGCACACCGCAGTAGATAAC |
| dm Cyt-c-p | Cytochrome c proximal | FBgn0284248 | CAATCTGCATGGTCTGATCG | GTGTCCTCGTTCCAGGTGAT |
| dm SdhB | Succinate dehydrogenase, subunit B (iron-sulfur), a protein of Complex II | FBgn0014028 | CCCGGACATGAACAACTTCT | CTCGACGGACTGCAGGTACT |
| dm Mt:CoI | Mitochondrial Cytochrome c oxidase subunit I (COX I), a protein of complex IV | FBgn0013674 | TTAGGATTGGCTGGAATACCT | TCATGTTGTGTAAGCATCTGG |
| dm Ets21C | Ets at 21C, human homology of NRF-2a, encodes a stress-inducible transcription factor | FBgn0005660 | CCAAACCGAACATGAACTACG | TTGTAGGCATACCGCTTTCC |
| dm Cdk4 | Cyclin-dependent kinase 4, qPCR primer | FBgn0016131 | GCCAGCAATCATGCGAATA | GCTCCACATGTTCGAAAACC |
| dm Cnc | Cap-n-collar (CncC) | FBgn0262975 | TTTTACCGACGAGGATCTGC | CCAGAACCGAGGAGTTGTTG |
| dm Opa1 | Optic atrophy 1 | FBgn0261276 | ATCTATCTGCCCGCTGCAC | GCGCAACTTGATGTCTACCA |
| dm Marf | Mitochondrial assembly regulatory factor (Mfn2) | FBgn0029870 | CCATGAGACGACCACCTTTA | CGCCACCTTGTACACATAGC |
| dm srl | spargel (dPGC1-alpha) | FBgn0037248 | ATGTAAAGGCCGATCCCTCT | CTGATGGTTCCCCAGTTGTT |
| dm Pink1 | PTEN-induced putative kinase 1 | FBgn0029891 | AATCAGGAACAAGAGCAGCA | TGATGTTTGAATTCGCTGGA |
| dm Hsp60A | Heat shock protein 60A | FBgn0015245 | GTGTCGAGACCACCAACGAG | GTTCTTGGCAATCGTCATGC |
| dm Hsp83 | Heat shock protein 83 (Hsp90) | FBgn0001233 | AGACGGCTGGTACTCTGACC | GCAATGGTTCCCAAGTTGTT |
| dm Drp1 | Dynamin related protein 1 | FBgn0026479 | GCTGATCTACAGCCCACTCG | CACTTCTTGGTGTGCAGGAA |
| dm AMPKa | AMP-activated protein kinase alpha subunit | FBgn0023169 | CTAAAGCTCTTCCGCCATCC | CCGCTCACGTACTCCATGAT |
| **Gene symbol** | **Gene name** | **FlyBase ID** | **Forward Primer Sequence** | **Reverse Primer Sequence** |
| dm Mt:CoI | Mitochondrial Cytochrome c oxidase subunit I (COX I), a protein of complex IV | FBgn0013674 | GCTGGGACAGGATGAACTGT | GAAGCTCCACCATGAGCAAT |
| dm Cdk4 | Cyclin-dependent kinase 4, mitochondrial copy number | FBgn0016131 | CTACGGTTCGGAGATGAAGC | ATGTCCACGGTGCTGTTGTA |

**Supplementary Figure 1**

(**A**) Lifespan of female flies fed a normal diet supplemented with 0.01% DMSO (Veh) or 500 µM BAM15 (BAM15). Veh N=276 and BAM15 N=333 flies included in analysis. Data are presented as the percentage of surviving flies per day and evaluated by log-rank test.

**Supplementary Figure 2**

(**A**) Percent of flies climbing fed a normal diet supplemented with 0.01% DMSO (Veh) or 500 µM BAM15 (BAM15) at 4 and 6 weeks of age. Panels A is presented as a box (mean±5-95% CI) and whiskers (minimum to maximum) plot with all individual points and was evaluated by unpaired Student’s t- test (Week 4: Veh N=12 vs. BAM15 N=15 biological replicates; N=10 flies per replicate, Week 6: Veh N=12 vs. BAM15 N=12 biological replicates; N=10 flies per replicate).

**Supplementary Figure 3**

(**A**) Illustration of substrate, uncoupler, inhibitor, titration protocol used for tandem high-resolution respirometry and fluorometry. Red indicates LEAK state, green indicates OXPHOS state, blue indicates ET state, and black indicates ROX state. Boxes represent the titration step. G3P: glycerol 3 phosphate, D: ADP, PM: pyruvate and malate, Pro: proline, F: FCCP, Rot: rotenone, and Ama: antimycin A. Representative tracing of (**B**) O2 flux and (**C**) H_2_O_2_ production in Veh-treated flies. Representative tracing of (**D**) O2 flux and (**E**) H_2_O_2_ production in Veh-treated flies.

**Supplementary Figure 4**

(**A**) Representative blot of AMPK phosphorylation (pAMPK^Thr172^), total AMPK expression, and Ponceau S. stain in thorax homogenates. (**B**) Quantification of pAMPK^Thr172^ expression relative to total AMPK normalized to Ponceau S. (**C**) AMPKα gene expression in thorax homogenates. Panels B-C are presented as are presented as a box (mean±5-95% CI) and whiskers (minimum to maximum) plot with all individual points. Panel B was assessed by Mann Whitney test (N=5 biological replicates; N=50 flies per replicate) and Panel C unpaired Student’s t-test (N=5 biological replicates; N=15 flies per replicate).
